# Proteomic Analysis of CHIKV-nsP3 Host Interactions in Liver Cells Identifies Novel Interacting Partners

**DOI:** 10.3390/ijms26146832

**Published:** 2025-07-16

**Authors:** Nimisha Mishra, Yash Chaudhary, Sakshi Chaudhary, Anjali Singh, Priyanshu Srivastava, Sujatha Sunil

**Affiliations:** 1 Vector Borne Disease Group, International Centre for Genetic Engineering and Biotechnology, New Delhi 110067, India; 2 TERI School of Advanced Studies (TERI-SAS), New Delhi 110070, India

**Keywords:** chikungunya virus (CHIKV), Huh7 cells, nonstructural protein 3, proteome, virus–host interactions

## Abstract

Chikungunya virus (CHIKV), a mosquito-borne alphavirus, has re-emerged, causing widespread outbreaks and a significant clinical burden. Despite advances in virology, the molecular mechanisms governing CHIKV’s interaction with host cells remain poorly understood. In this study, we aimed to identify novel host protein interactors of the CHIKV nonstructural protein 3 (nsP3), a critical component of the viral replication complex, using mass spectrometry-based proteomic profiling in liver-derived Huh7 cells. Co-immunoprecipitation followed by LC-MS/MS identified a wide array of host proteins associated with nsP3, revealing 52 proteins classified as high-confidence (FDR of 1%, and unique peptides > 2) CHIKV-specific interactors. A bioinformatic analysis using STRING and Cytoscape uncovered interaction networks enriched in metabolic processes, RNA processing, translation regulation, cellular detoxification, stress responses, and immune signaling pathways. A subcellular localization analysis showed that many interactors reside in the cytosol, while others localize to the nucleus, nucleolus, and mitochondria. Selected novel host protein interactions were validated through co-immunoprecipitation and immunofluorescence assays. Our findings provide new insights into the host cellular pathways hijacked by CHIKV and highlight potential targets for therapeutic intervention. This is the first report mapping direct nsP3–host protein interactions in Huh7 cells during CHIKV infection.

## 1. Introduction

Viruses rely on host cell machinery to ensure their efficient replication and infection. They hijack the cellular and metabolic processes to modulate the host system and create an optimal environment for their life cycle. By leveraging host proteins and evading immune responses, they ensure efficient genome replication and virus production in susceptible cells [1].

Chikungunya virus (CHIKV), an enveloped, positive-sense single-stranded RNA virus of the *Togaviridae* family and Alphavirus genus, is a significant global health concern due to its capacity to cause widespread outbreaks [2]. Transmitted by Aedes mosquitoes, the acute phase of CHIKV infection is marked by high fever, severe arthralgia, rash, and myalgia, often resolving within weeks. However, a substantial proportion of patients, estimated at up to 25%, experience chronic joint pain persisting for months or years, severely impacting quality of life [3,4]. While the primary pathological manifestations of CHIKV infection are musculoskeletal, systemic effects are frequently reported [5]. Among these, hepatic involvement has been observed in some patients, typically as elevated liver enzymes—alanine aminotransferase (ALT) and aspartate aminotransferase (AST)—and, in severe cases, histopathological evidence of hepatic inflammation [6,7]. However, these manifestations are more likely attributable to systemic inflammation or immune-mediated effects than direct infection of hepatocytes, for which current evidence remains limited.

It is noteworthy, however, that the liver plays a central hub in orchestrating metabolic and immunological responses to viral infections, and it remains an important site for systemic viral pathogenesis [8,9]. Its susceptibility to CHIKV suggests that the virus may exploit hepatic cells to support replication or evade immune defenses, potentially contributing to systemic disease progression. Despite this, research on CHIKV–host interactions has predominantly focused on musculoskeletal tissues and immune cells, leaving a critical gap in understanding liver-specific pathogenesis [5,10,11].

The CHIKV genome, approximately 11.8 kb in length, encodes four nonstructural proteins (nsP1–nsP4) that orchestrate viral replication and manipulate host cellular processes. Among these, nonstructural protein 3 (nsP3) stands out as a multifunctional regulator with roles in RNA synthesis, host immune suppression, suppression of host RNAi, and cellular stress modulation [12]. Structurally, nsP3 comprises three domains: the macrodomain, the alphavirus unique domain (AUD), and the hypervariable domain (HVD). The HVD, an intrinsically disordered region, serves as a dynamic interaction hub, binding host proteins to facilitate viral survival and replication [13]. A well-characterized interaction involves nsP3 recruiting G3BP1 and G3BP2 to inhibit stress granule assembly, a host antiviral mechanism that sequesters viral RNA, thereby promoting replication [14]. Other reports have suggested the interaction of the CHIKV-nsP3 protein with the NAP1 protein, which plays a crucial role in viral pathogenesis [15,16].

To investigate CHIKV–host interactions in a human liver-derived cellular context, we employed the Huh7 hepatoma cell line—a widely used model in virus infection studies, primarily owing to its permissiveness to RNA viruses and suitability for several downstream validation analyses [17,18]. The present study was undertaken to systematically identify novel interacting partners of CHIKV-nsP3 in Huh7 cells. We investigated the probable host-interacting factors by performing co-immunoprecipitation of CHIKV-infected human hepatic Huh7 cells with recombinant nsP3, followed by mass spectrometry (MS). The interactions between the viral nsP3 protein and host cellular partners were subsequently validated through protein–protein interaction assays. Our findings provide novel insights into the host factors associated with CHIKV-nsP3, highlighting their critical roles in viral pathogenesis during CHIKV infection.

## 2. Results

### 2.1. Mass Spectrometry Identifies Distinct Host-Interacting Partners of the CHIKV-nsP3 Protein

**Figure 1:**
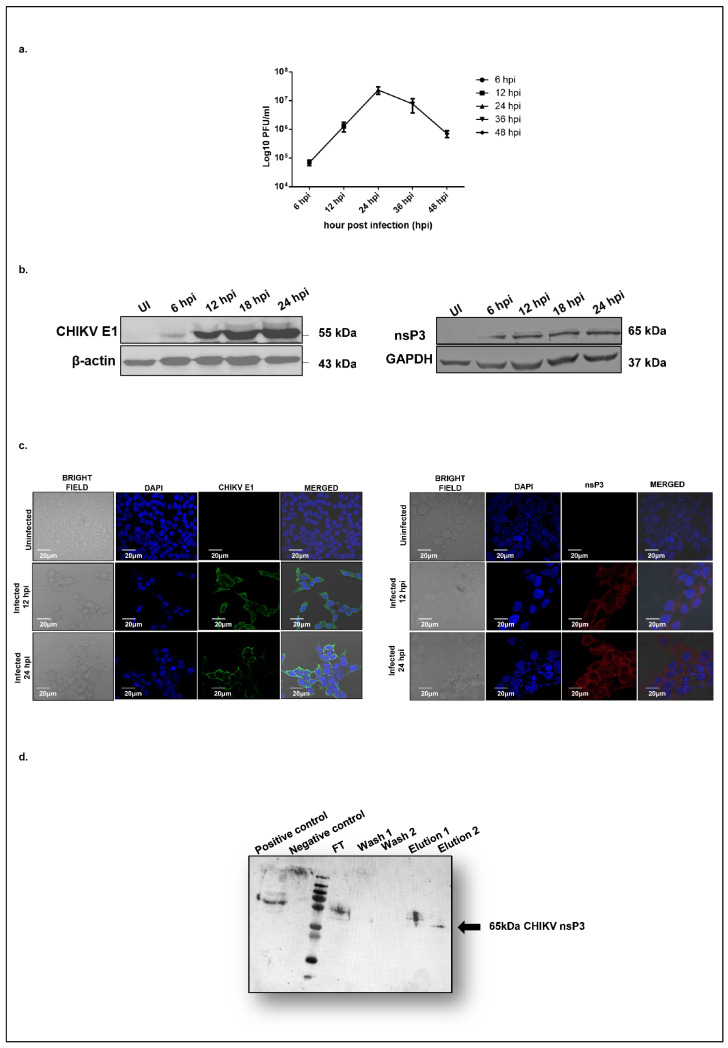
Co-Immunoprecipitation of CHIKV-nsP3 interacting partners in liver-derived Huh7 cells. (**a**) A plaque assay was performed to estimate the change in the virus titer in the culture filtrate of Huh7 cells during the time course of infection at 6, 12, 24, 36, and 48 hpi. (**b**) Western blot depicting the change in the expression pattern of CHIKV E1 and CHIKV-nsP3 protein at 6, 12, 18, and 24 hpi where uninfected cell lysate served as a control. (**c**) The immunofluorescence assay shows the distribution and expression of CHIKV E1 and CHIKV-nsP3 proteins in infected Huh7 cells at 12 and 24 hpi. Uninfected cells were used as a control. Nuclei are stained with DAPI (blue), and CHIKV E1 protein with Alexa Fluor 488 (green) and CHIKV nsP3 protein with Alexa Fluor 594 (red). scale bar, 10 μm. (**d**) Immunoblot detection of the CHIKV-nsP3 protein at an expected molecular weight of 65 kDa in Co-IP elutions.

To characterize the dynamics of CHIKV replication in hepatocyte-derived cells, we assessed viral replication in Huh7 cells over 48 h post-infection using a plaque assay. At a multiplicity of infection (MOI) of 1, CHIKV exhibited a logarithmic growth phase beginning at 12 h post-infection (hpi), reached maximal titers at 24 hpi, and declined by 36 hpi (Figure 1a). To further investigate viral protein expression, we profiled the temporal expression of the CHIKV nonstructural protein 3 (nsP3) and the structural envelope protein E1 at the same time points. A Western blot analysis revealed that both E1 (Figure 1b, left panel) and nsP3 (Figure 1b, right panel) became detectable at 12 hpi, with expression levels peaking at 24 hpi (Figure 1b). These findings were corroborated by confocal microscopy, which confirmed the cytosolic localization of both CHIKV-nsP3 and E1 in infected Huh7 cells (Figure 1c). Based on these observations, 12 hpi was selected as the optimal time point for downstream proteomic profiling of CHIKV-nsP3 interacting partners. We hypothesized that this time point likely marks the onset of robust viral protein synthesis and corresponds to the early phase of active viral replication, and thereby would provide the best opportunity to capture those host proteins interacting with CHIKV-nsP3 to regulate viral infection.

To identify those host proteins interacting with CHIKV-nsP3, immunoprecipitation coupled with the LC/MS (IP LC/MS) approach was used for our study. Huh7 cells were infected with CHIKV at an MOI of 1, and uninfected cells were maintained as controls. Cells were harvested at 12 hpi and lysed using a Co-IP lysis buffer. Prior to Co-IP, the specificity of the nsP3 antibody was validated in the CHIKV-infected Huh7 cells (Supplementary Figure S1) and further confirmed in immunoprecipitated elutes (Figure 1d). Three independent biological replicates of LC-MS/MS analyses from all isolated protein complexes enabled the identification of 16,501 peptides from 2829 proteins with a false discovery rate (FDR) of 1%. The CHIKV-nsP3 protein was identified with 22 unique peptides in the infected samples, achieving 46% sequence coverage and a high confidence score of 119.82. Other viral proteins that were detected in the mass spec analysis of infected replicates are nsP2 (4 peptides, score 9.56), envelope E1 (6 peptides, score 9.87), and capsid (12 peptides, score 44.3). Notably, no CHIKV-derived peptides were detected in the uninfected controls. A complete list of all the host proteins identified through the LC-MS/MS with significant scores, sequence coverage, and peptide sequences and numbers is shown in Supplementary Table S1. To identify the cellular proteins associated with CHIKV-nsP3, a dataset was used to query a human protein database. A total of 52 high-confidence host proteins were identified exclusively in infected cells. Stringent criteria, including detection in all three biological replicates, FDR ≤ 1%, and identification with more than two unique peptides per protein, were used to derive the list of host proteins. The final list of 52 host-interacting proteins of CHIKV-nsP3 identified through mass spectrometry is presented in Table 1.

### 2.2. CHIKV-nsP3 Interacts with the Host Metabolic Pathway Proteins and Translational Regulators

**Figure 2:**
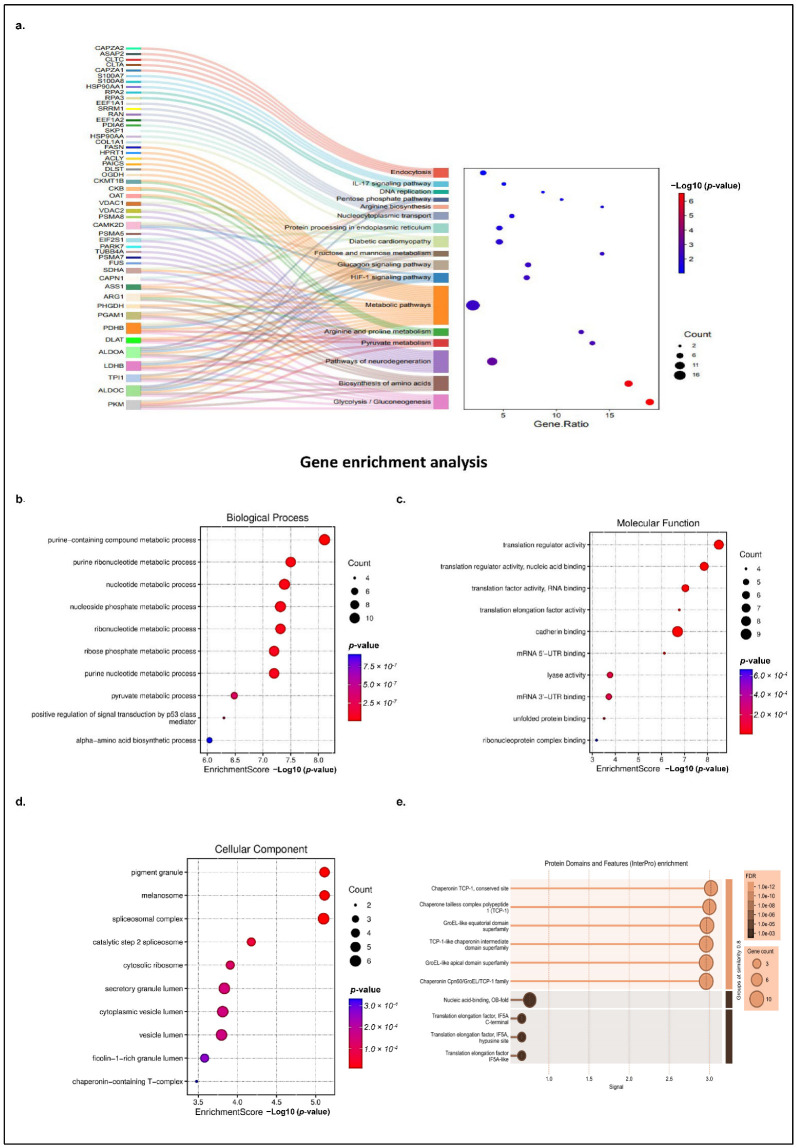
Pathway and gene ontology enrichment analysis of the CHIKV-nsP3 interacting partner: (**a**) Sankey and the dot plot of the core metabolic proteins and their pathways, with the cellular processes involved plotted with the help of SRplots (SRplot: a free online platform for data visualization and graphing). (**b**) GO enrichment analysis of crucial biological processes associated with CHIKV-nsP3 interacting proteins. The color of the circles denotes the enrichment significance *p*-value, and the size of the circle represents the number of genes in the functional category. (**c**) GO enrichment analysis of the molecular functions associated with CHIKV-nsP3 interacting proteins. (**d**) GO enrichment analysis of the cellular components of the CHIKV-nsP3 interacting proteins. (**e**) The protein domain associated with the host proteins based on the maximum FDR value ≤ 0.05 and minimal signal strength ≥ 0.01 using STRING 12.0.

Next, the identified host proteins were subjected to a comprehensive pathway analysis using a suite of software tools, such as KOBAS (v3.0), and the databases used for pathway analysis include KEGG and Reactome (Reactome Pathway Database; accessed on 3 March 2025) web server (KOBAS; v3.0; accessed on 3 March 2025). The results revealed statistically significant pathways (with corrected *p*-value ≤ 0.05), linked to cellular metabolism, translation regulation, neurological disease, diabetes neuropathy, immune signaling, spliceosome, endocytosis, and nucleocytoplasmic processing, as shown in Figure 2a. Notably, pathways associated with host cell metabolism formed the largest cluster (Figure 2a, highlighted in orange) comprising 27 proteins, suggesting that CHIKV hijacks the host metabolic machinery for its replication and energy requirement. Among the most enriched were metabolic and biosynthetic pathways, including the TCA cycle, ribosome (22 proteins), glycolysis/gluconeogenesis (9 proteins), and biosynthesis of amino acids (9 proteins), highlighting the potential manipulation of host metabolism and protein synthesis machinery by nsP3. Several proteins, including PKM, ALDOA, LDHB, PDHB, DLAT, and PDHA, which are key components of glycolysis, gluconeogenesis, and the TCA cycle, were found to be enriched, suggesting a viral strategy to reprogram host metabolism for efficient replication. Similarly, proteins such as ASS1, ARG1, and PHGDH that were involved in amino acid biosynthesis and arginine/proline metabolism were also found to be enriched, indicating the potential modulation of nitrogen balance and amino acid availability (Figure 2a, highlighted in brown and green).

Interestingly, several disease-related pathways were significantly enriched, including those associated with neurodegeneration (e.g., Parkinson’s, Huntington’s, and Alzheimer’s diseases), diabetes neuropathy, and immune-related signaling, such as the HIF-1 and glucagon signaling pathways. The identified proteins that are known to be associated with disease progression, such as transglutaminase2 (TGM2), and VDAC1 and 2, are responsible for Parkinson’s, Huntington, and Alzheimer’s neurological disorders, emphasizing the critical importance of neurological complications linked to CHIKD (Figure 2a, highlighted in purple color). Additionally, proteins such as VDAC1, VDAC2, and CKMT1B, which are associated with mitochondrial function and energy transport, were enriched in pathways like diabetic cardiomyopathy (Figure 2a, light green color) and metabolic pathways, highlighting the impact of nsP3 on mitochondrial dynamics and cellular energetics. Immune signaling-related proteins such as CAPZA2, HSP90AA, and CAMK2D were mapped to IL-17, HIF-1, and glucagon signaling pathways, which are involved in inflammatory and stress responses (Figure 2a, dark blue, light blue, and light brown). These findings may reflect the conserved cellular mechanisms that are hijacked by the virus to facilitate replication or modulate the host immune response. These data also suggest that nsP3 interacts with a diverse set of host proteins involved in fundamental cellular processes, stress responses, and disease-relevant signaling, underlining its central role in CHIKV pathogenesis.

Several other host proteins were also analyzed, such as proteins involved in transport and stress response, including HSPA8, HSPA1A, PSMA6, and PSMA7, which were linked to the proteasome and endoplasmic reticulum protein processing pathways, pointing to the viral manipulation of host protein quality control systems (Figure 2a, light green color). Moreover, nuclear and transport-related proteins like RPA2, EIF4A1, CLTC, and NUPs were enriched in nucleocytoplasmic transport, spliceosome, and DNA replication pathways, suggesting that nsP3 may interfere with nuclear-cytoplasmic trafficking and host gene regulation (Figure 2a, light purple color).

Apart from this, proteins involved in translational regulation, primarily initiation and elongation factors such as EEF1A2, EEF1G, EIF5A, and EIF2S3, were also prominent, indicating that CHIKV relies heavily on the host ribosomal machinery for viral protein synthesis, potentially disrupting cellular innate immune defenses. Furthermore, mRNA splicing-related proteins, including splicing factor 3a and 3b, small nuclear ribonuclear protein (SNRPD3), SNRPN, SNRPA1, and SF3A2, were also identified among the cluster, suggesting that CHIKV manipulates the host spliceosome to alter cellular gene expression, optimizing the production of specific spliced viral products during infection. The CHIKV-nsP3 protein was also found to coprecipitate with several cellular proteins involved in ribosome biogenesis, such as RNA helicase DDX17 and the rRNA methyltransferase fibrillarin.

The identified proteins demonstrate that nsP3 interacts with key nodes of host cellular function, including metabolism, protein homeostasis, signaling, and gene expression regulation, reflecting the virus’s capacity to hijack and modulate host pathways to facilitate its replication and persistence.

A Gene Ontology enrichment analysis of the selected proteins revealed crucial biological processes involved, the regulatory molecular functions of the host proteins, associated cellular components, and the protein domains involved. The top 10 key biological processes involved were related to metabolic processes, such as nucleotide synthesis (*p* value 4.09873 × 10^−8^), the pyruvate metabolic process (*p* value 3.27629 × 10^−7^), and the amino acid metabolic process (*p* value 9.16788 × 10^−7^). (Figure 2b). An enrichment analysis accurately assigned key molecular functions of the host-interacting proteins, including translation regulation (*p* value 3.22709 × 10^−9^), mRNA 5′-UTR binding (*p* value 7.49673 × 10^−7^), lyase activity (*p* value 0.000171239), and RNA binding (*p* value 0.000791514) (Figure 2c). The detection of additional molecular functions other than the top 10 shown in the Figure 2c, such as peroxidase activity (*p* value 0.006140365), indicated its role in cellular oxidative stress responses during infection. In agreement with the biological process and molecular functions, a GO functional enrichment analysis of the identified host proteins also showed that most of the interacting partners are a major component of the ribosomal compartment (*p* value 1.23555 × 10^−4^), spliceosome machinery (*p* value 7.9272 × 10^−6^), cytoplasmic vesicle (*p* value 1.54373 × 10^−4^), and Chaperone complex (*p* value 3.33628 × 10^−4^) (Figure 2d). An analysis also revealed that the localization of nsP3 interacting host partners is somewhat consistent with the localization of viral protein, nsP3, i.e., cytosol (previously shown in Figure 1c).

Moreover, the host proteins were further examined for the enriched protein domains using GO term enrichment, and it was found that the Chaperonin TCP-1 (FDR 7.53 × 10^−12^), GroEL-like equatorial domain superfamily (FDR 7.53 × 10^−12^), Nucleic acid-binding (FDR 0.00090), and translation elongation factor IF5A C-terminal (FDR 0.0047) were among the top 10 significantly enriched (Figure 2e).

Taken together, an in-depth analysis of the interacting partners reveals that CHIKv-nsP3 interacts with the host proteins involved in metabolic pathways and translational regulators.

### 2.3. Host Interacting Partners of CHIKV-nsP3 Form a Highly Interconnected Regulatory Network

**Figure 3:**
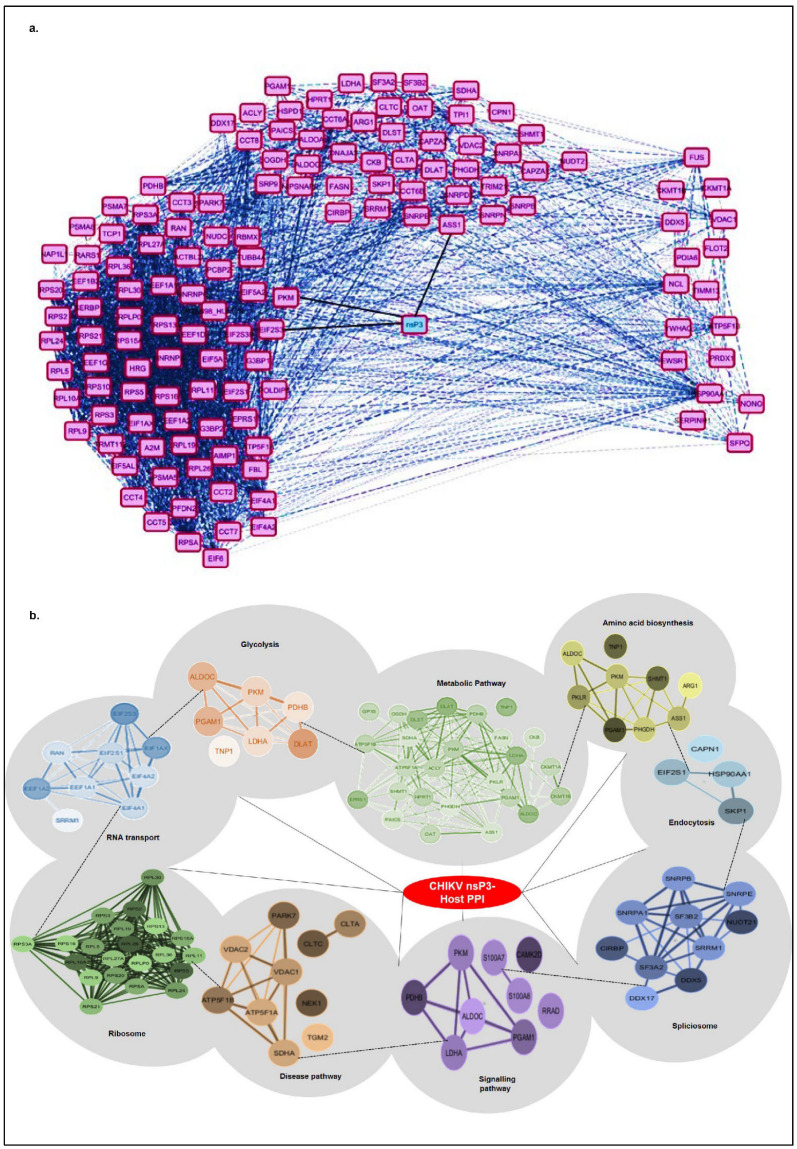
CHIKV-nsP3 host protein–protein interaction network derived from Co-IP and LC-MS/MS-based studies. (**a**) This network visualizes host proteins that interact directly or indirectly with the chikungunya virus (CHIKV) nonstructural protein 3 (nsP3), identified through Co-IP experiments in Huh7 human hepatoma cells. The central, cyan-colored node represents nsP3, while magenta nodes represent host proteins. Solid black edges indicate novel interactions from the current study between nsP3 and host proteins detected with high confidence. Dashed grey edges denote additional host–host interactions mapped from known protein–protein interaction databases (STRING). The dense connectivity on the left indicates ribosomal and translation-related factors clustering, suggesting that CHIKV nsP3 may interact with or co-opt host translation machinery. Nodes on the right are more loosely connected and may represent nuclear or stress granule-associated proteins. The network layout was optimized for visualization clarity. (**b**) Pathway target networks (P-T network): A network constructed from significant pathway enrichment results connecting different human protein pathways with the CHIKV-nsP3 protein present at the center. The significant pathways are present in the form of different clusters (around the CHIKV-nsP3 protein). The pathways are connected with the help of dashed edges, while the 8 edges in the centre connect with the virus CHIKV-nsP3 protein. The host proteins are present in the form of colored nodes according to their significant pathways. While the edges represent the strength of interaction (the thicker edges represent significant interactions amongst the proteins). The networks were obtained with default settings for a full STRING network, displaying edges in high-confidence mode with a minimum interaction score of 0.7; a maximum FDR ≤ 0.05; and a minimum strength ≥ 0.01 and displaying no disconnected nodes.

To investigate the functional and physical associations of host proteins interacting with CHIKV-nsP3, we conducted a protein–protein interaction (PPI) analysis using STRING 12 and visualized the network in Cytoscape, applying a high confidence threshold of 0.7 (Figure 3a,b). The interactome map (Figure 3a) positions CHIKV-nsP3 as a central cyan node, surrounded by magenta nodes representing human host proteins identified via co-immunoprecipitation (Co-IP) assays. Solid black edges denote direct interactions between nsP3 and host proteins, while dashed grey edges indicate known protein–protein associations among host proteins, sourced from curated databases like STRING, providing functional context and clustering information. The network reveals distinct functional modules: ribosomal proteins, translational initiation factors, and RNA-binding proteins presented at the left of nsP3, while proteins involved in nuclear transport, stress granules, and RNA metabolism presented to the right. The PPI network organization suggests nsP3′s multifaceted roles in modulating host translation, RNA processing, and potentially subverting innate immune signaling pathways, thereby facilitating viral replication and immune evasion. The dense interconnectivity within clusters reflects functional co-regulation and suggests that nsP3 may act as a central hub in rewiring host cellular pathways to favor viral replication.

To further dissect the biological relevance of these interactions, we generated a pathway-based network (Figure 3b) based on the significant pathways presented in Table 2. The crucial pathways that were defined in the network belong to metabolism, ribosomal proteins, transport, spliceosome, neurodegeneration, endocytosis, and immune signaling. We excluded a few pathways, such as ferroptosis, that were not well-represented in PPI using STRING. CHIKV-nsP3 remains centrally located, with peripheral clusters connected by dashed edges indicating inter-cluster interactions. The largest cluster, associated with metabolic pathways, comprises 27 nodes (light green) and ribosomal protein folding (dark green, densely interconnected, consisting of 22 proteins). Smaller clusters include proteins involved in amino acid biosynthesis (yellow), glycolysis/gluconeogenesis (orange), translation factors and transport proteins (blue), the spliceosome (blue), neurodegeneration pathways (brown), endocytosis (light blue), and immune signaling (purple), all critical for regulating virus–host interactions. The high degree of connectivity within and between clusters indicates a coordinated host response, likely exploited by CHIKV for replication and protein trafficking. This intricate network also suggests functional interdependence, with nsP3 potentially interacting with entire protein complexes rather than individual proteins.

Collectively, these findings underscore the complex mechanisms by which CHIKV-nsP3 co-opts host metabolic, translational, and cellular processes to enhance viral replication and pathogenesis. The dense interconnectivity within the network highlights nsP3′s role as a central hub in rewiring host cellular pathways to favor viral propagation.

### 2.4. Confocal Microscopy Reveals Subcellular Distribution of nsP3-Interacting Host Proteins

**Figure 4:**
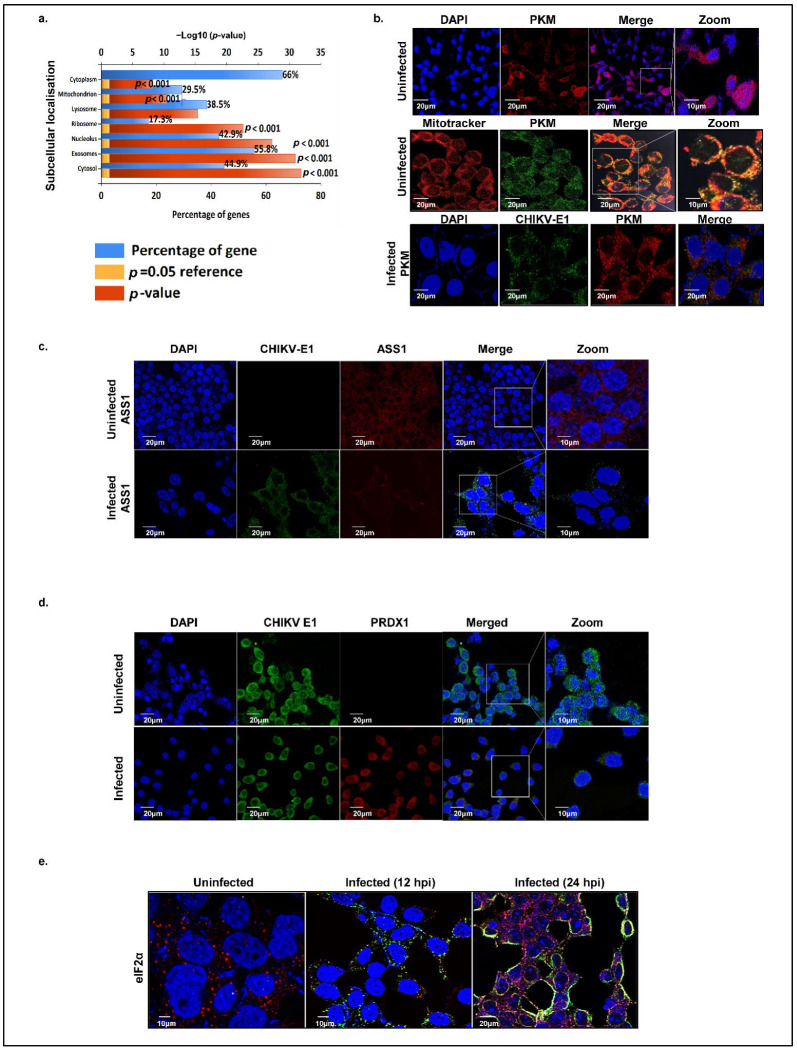
Subcellular localization of the CHIKV-nsP3 host-interacting partners based on in silico prediction and in vitro validation. (**a**) The bar graph represents the percentage (%) of host proteins localized in the different cellular compartments. (**b**) Immunofluorescence showing the expression pattern of PKM in different cellular compartments in an uninfected state and an infected state. Nuclei are stained with DAPI (blue) and mitochondria with mitotracker (Red), PKM protein with Alexa Fluor 488 (green); scale bar, 20 μm (**c**,**d**). Immunofluorescence showing the expression pattern of ASS1 and PRDX1 in uninfected and infected conditions. Nuclei are stained with DAPI (blue) and CHIKV E1 protein with Alexa Fluor 488 (green); scale bar, 20 μm (**e**) Immunofluorescence showing the temporal expression pattern of eIF2α in an uninfected state and distribution of eIF2α during infection at 12 hpi and 24 hpi (at MoI of 1.0 in Huh7 cells) in different cellular compartments. Immunostaining was carried out with antibodies against viral protein (green) and eIF2α (red) and respective Alexa Fluor 488 (mice) and Alexa Fluor 594 (rabbit) secondary antibodies. The nucleus was visualized with DAPI (blue). Images were taken using the confocal microscope.

Efficient viral replication hinges on the transport of signaling molecules and the modulation of functional sites in response to infection, processes critically dependent on the precise and timely localization of both host and viral proteins. To explore the dynamics of the localization of the host proteins interacting with CHIKV-nsP3, we analyzed the enrichment of cellular compartments for the top hit predicted subcellular proteins using FunRich and Deeploc 2.0. These proteins were broadly classified into the cytoplasmic, nuclear, nucleolar, mitochondrial, ribosomal, and extracellular matrix/secretory compartments (Figure 4a). Our analysis revealed that about 66% of the cellular proteins were localized in cytoplasm, while 42.9% proteins were nucleolar, suggesting significant cytoplasmic—nuclear shuttling of these host proteins during viral replication. We identified nuclear proteins with known innate immunity-related functions, such as FBL, NCL, DDX17, and HSP40, which may translocate to the cytoplasm to modulate immune signaling, suggesting that CHIKV manipulates host defenses by redistributing specific nuclear proteins. Further, 55.9% of the proteins localized to exosomes, highlighting the role of extracellular vesicles in the CHIKV pathogenesis. Mitochondrial proteins accounted for 29.5% of nsP3 interactors, reflecting viral disruption of mitochondrial dynamics and metabolism to evade immunity. Furthermore, 38.5% of proteins localized to lysosomes and 17.3% to ribosomes.

To validate in silico predictions of host protein localization during uninfected and CHIKV-infected states, we employed confocal microscopy to examine some of the key interacting host proteins, namely, argininosuccinate synthase 1 (ASS1), pyruvate kinase M (PKM), eukaryotic translation initiation factor alpha subunit (eIF2α), and peroxiredoxin 1 (PRDX1). CHIKV E1 protein was used as a marker to confirm the infection status within cells. We first focused on the subcellular localization of pyruvate kinase M (PKM), evaluating its co-localization with mitochondria using MitoTracker Red and with nuclei stained by DAPI. In uninfected cells, PKM was observed in the cytosol, mitochondria, and nucleus (Figure 4b, first and second panels), whereas at 12 hpi, it was restricted to the cytosol (Figure 4b, third panel). Next, we examined ASS1 in Huh7 cells, finding it consistently localized to the cytosol in both uninfected and infected conditions (Figure 4c). Peroxiredoxin was similarly confined to the cytosol under both conditions. However, a low expression of peroxiredoxin 1 was also seen in the nucleus during the uninfected condition (Figure 4d). A temporal localization analysis of host translation initiation factor eIF2α at 12 hpi and 24 hpi revealed a dynamic subcellular redistribution of the protein. At 12 hpi, eIF2α was predominantly localized in the cytosol (Figure 4e); however, by 24 hpi, the protein exhibited a marked translocation to the nucleus, with a concomitant reduction in cytosolic presence. This temporal shift in eIF2α localization suggests a virus-induced modulation of host translational control mechanisms, potentially contributing to the cellular stress response during CHIKV replication.

While the steady-state subcellular localization of the above host proteins is well-documented, our confocal imaging data provide insights into the subcellular spatial and temporal redistribution of these proteins upon CHIKV infection.

### 2.5. Immunoprecipitation Validates the Interaction of Host Partners with CHIKV-nsP3

To confirm the specificity of host proteins’ interactions identified through Co-IP-based mass spectrometry and to strengthen the reliability of our dataset, we performed independent validation using immunoprecipitation (IP) and confocal microscopy-based co-localization assays. The selected host factors, ASS1, PKM, eIF2α, and PRDX1, were assessed with a CHIKV-infected MOI of 1 in Huh7 cell lysates collected at 12 hpi. These validation experiments provided direct evidence of physical association and spatial co-localization with nsP3 during the early stages of infection.

### 2.6. Validation of Interaction Between Host Partner PKM and CHIKV-nsP3

**Figure 5:**
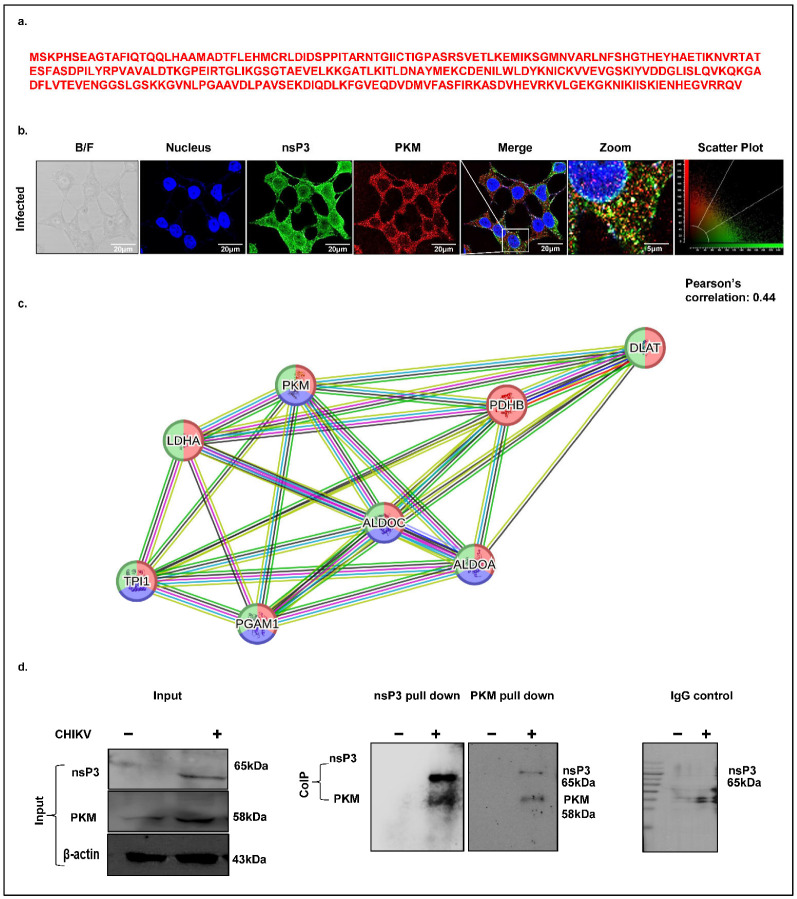
CHIKV-nsP3 protein interacts with PKM. (**a**) Peptide sequence of PKM identified through mass spectrometry. (**b**) IFA showed colocalization of PKM with CHIKV-nsP3 at 12 hpi (at MoI of 1.0). Immunostaining was carried out with antibodies against CHIKV-nsP3 (green) and PKM (red) and respective Alexa Fluor secondary 488 (mice) and Alexa Fluor 594 (rabbit) secondary antibodies. The nucleus was visualized with DAPI (blue). Images were taken using the confocal microscope. A scale bar of 20 μm was used. (**c**) The STRING network represents the host proteins involved in pyruvate metabolism. Nodes represent the host proteins, and edges represent the association between the proteins based on similarity in functions. Filled nodes denote the prediction of the 3D structure of the specific protein. (**d**) Immunoblotting image showing the direct interaction between PKM and CHIKV-nsP3 during infection. Uninfected and infected Huh7 cells cell lysates were prepared 12 h post-infection and used for the Co-IP experiment. The first immunoblot represents 10% of the total cellular lysate (input) used for the pull-down experiment and Western blotting, and β-actin was used as a loading control. The second and third immunoblots show the interaction of CHIKV nsP3 protein with PKM using both PKM and anti-CHIKV nsP3 antibodies. The fourth immunoblot represents the negative control, where rabbit IgG was used to immunoprecipitate the protein complex and was probed with anti-nsP3 antibody.

Virus-infected cells often exhibit elevated carbon metabolism to support viral replication or fuel host antiviral responses. Pyruvate kinase M (PKM), a key glycolytic enzyme catalyzing the conversion of phosphoenolpyruvate (PEP) to pyruvate and ATP, was identified as a high-confidence CHIKV-nsP3 interacting protein in our mass spectrometry dataset, with 19 unique peptides detected (Figure 5a). To validate this interaction, we first examined the spatial distribution of PKM and nsP3 in CHIKV-infected Huh7 cells (MOI 1, 12 hpi) using immunofluorescence. Confocal microscopy revealed the cytosolic co-localization of nsP3 and PKM, with a Pearson’s correlation coefficient of 0.44, indicating a moderate degree of spatial overlap (Figure 5b). Next, we generated a STRING network of the pyruvate metabolism pathway, which revealed a significant protein–protein interaction (PPI) enrichment, comprising 8 nodes and 20 edges. The network included key glycolytic enzymes identified in our proteomic screen, such as PKM, TPI1, ALDOA, and ALDOC, highlighted by high-confidence annotations from KEGG, Reactome, and Wiki Pathways with a signal strength of 1.93 and an FDR of 6.40 × 10^−9^ (Figure 5c). Finally, to confirm a direct physical interaction between PKM and nsP3, we performed co-immunoprecipitation (Co-IP) assays using CHIKV-infected Huh7 lysates collected at 12 hpi. An immunoblot analysis confirmed the expression of PKM and nsP3 at their expected molecular weights (58 kDa and 65 kDa, respectively), with uninfected lysates serving as negative controls (Figure 4d, first blot). The Co-IP results demonstrated a robust interaction between PKM and nsP3, validating our mass spectrometry findings (Figure 5d, second and third blots), with IgG serving as a negative control (fourth blot).

### 2.7. Validation of Interaction Between Host Partner ASS1 and CHIKV-nsP3

**Figure 6:**
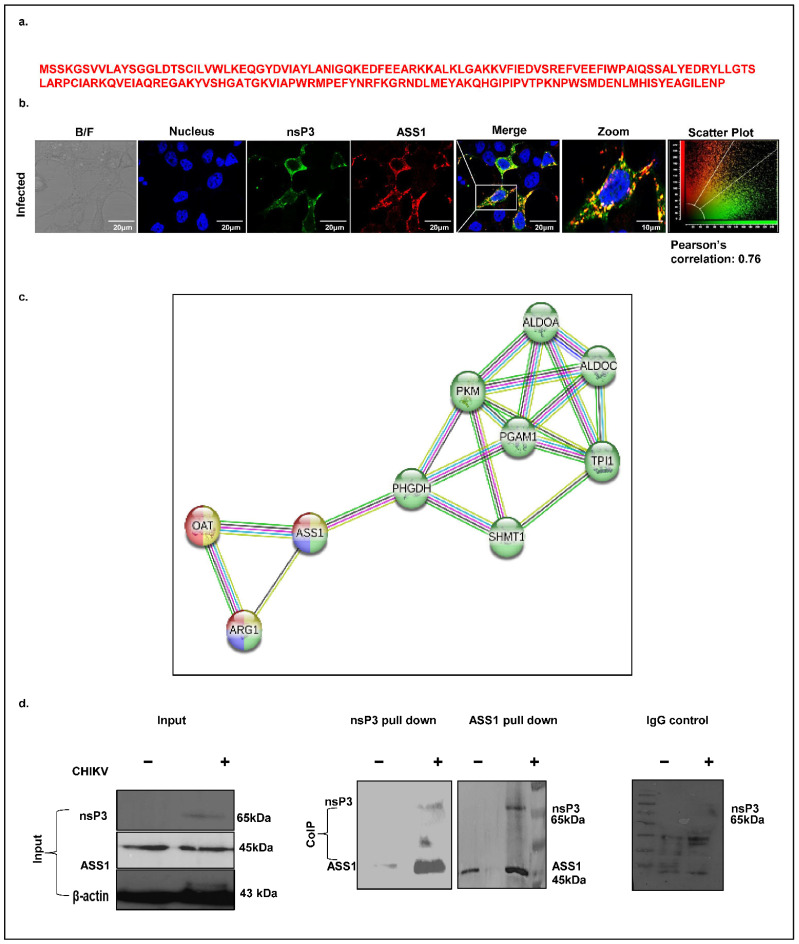
CHIKV-nsP3 protein interacts with ASS1. (**a**) Peptide sequence of ASS1 identified through mass spectrometry. (**b**) IFA showed colocalization of ASS1 with CHIKV-nsP3 at 12 hpi (at MoI of 1.0). Immunostaining was carried out with antibodies against CHIKV-nsP3 (green) and ASS1 (red) and respective Alexa Fluor secondary 488 (mice) and Alexa Fluor 594 (rabbit) secondary antibodies. The nucleus was visualized with DAPI (blue). Images were taken using the confocal microscope. A scale bar of 20 μm was used. (**c**) The STRING network represents the host proteins involved in Amino acid biosynthesis and metabolism. Nodes represent the host proteins, and edges represent the association between the proteins based on similarity in functions. Filled nodes denote the prediction of the 3D structure of the specific protein. Light blue and purple edges represent the known interactions that have been experimentally validated, whereas green, red, and dark blue denote the predicted interactions. (**d**) Immunoblotting image showing the direct interaction between ASS1 and CHIKV-nsP3 during infection. Uninfected and infected Huh7 cells cell lysates were prepared 12 h post-infection and used for the Co-IP experiment. The first immunoblot represents 10% of the cellular lysate (input) used for Western blotting, and β-actin was used as a loading control. The second and third immunoblots are the co-immunoprecipitation analysis showing the interaction of CHIKV-nsP3 protein with ASS1 using the nsP3 and ASS1 antibodies. The fourth immunoblot represents the negative control, where rabbit IgG was used to immunoprecipitate the protein complex and was probed with the anti-nsP3 antibody.

ASS1, a key enzyme in arginine biosynthesis, has previously been implicated in viral infections such as HSV, where its depletion promotes viral replication. However, its role in alphavirus infections, including CHIKV, remains unexplored. Our mass spectrometry analysis identified ASS1 as a potential CHIKV-nsP3 interacting host protein, supported by the detection of four unique peptides (Figure 6a). To investigate this interaction, we first assessed the spatial localization of ASS1 and nsP3 in CHIKV-infected Huh7 cells (MOI 1, 12 hpi) using confocal microscopy. A co-localization analysis revealed strong cytosolic overlap between the two proteins, with a Pearson correlation coefficient t of 0.76 (Figure 6b). Next, we performed a STRING-based protein–protein interaction (PPI) analysis at high confidence (interaction score ≥ 0.7). This revealed a significantly enriched network comprising 10 nodes and 19 edges, with the involved proteins mapping primarily to amino acid biosynthesis pathways with an FDR of 2.65 × 10^−28^ and a signal strength of 3.28 (Figure 6c). KEGG, Reactome, and WikiPathways analyses consistently identified arginine metabolism as the most significantly enriched biological process (FDR values: KEGG, 3.45 × 10^−5^; Reactome, 0.0044; WikiPathways, 3.45 × 10^−5^), with key components including ASS1, Arginase1, and OAT. Finally, to validate the physical interaction between ASS1 and nsP3, we performed co-immunoprecipitation in CHIKV-infected Huh7 cell lysates harvested at 12 hpi. Immunoblotting confirmed that ASS1 associates with nsP3 during CHIKV infection (Figure 6d), supporting its potential role in virus–host metabolic interactions.

### 2.8. Validation of Interaction Between Host Partner PRDX1 and CHIKV-nsP3

**Figure 7:**
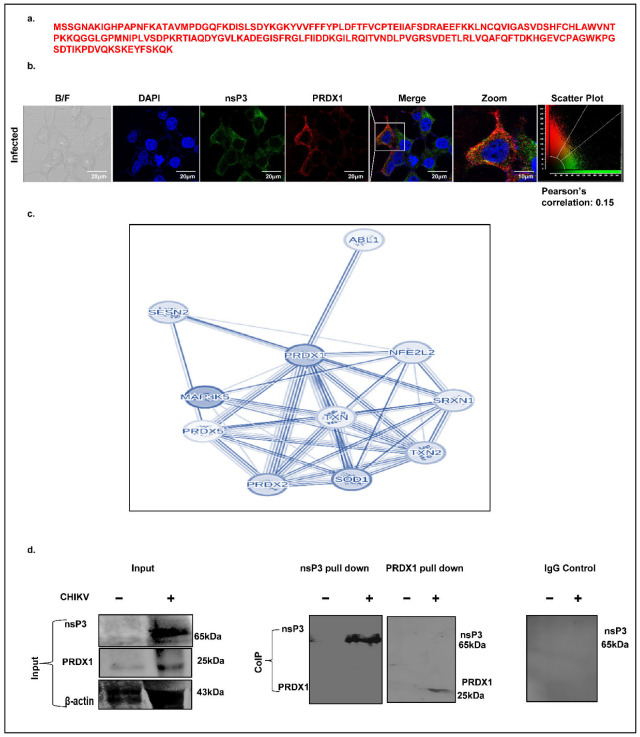
CHIKV-nsP3 protein interacts with PRDX1. (**a**) Peptide sequence of PRDX1 identified through mass spectrometry. (**b**) Immunofluorescence showing the colocalization of PRDX1 with CHIKV-nsP3 (at MoI of 1.0 in Huh7 cells); scale bar 20 μm used. (**c**) The STRING network represents the host proteins involved in host cell peroxidase activity. (**d**) Immunoblotting image showing the interaction between PRDX1 and CHIKV-nsP3 during infection. The first blot represents the total cell lysate used for the pull-down experiment (input), where β-actin served as a loading control. The second and third immunoblot is the co-immunoprecipitation analysis showing the interaction of CHIKV-nsP3 protein with PRDX1 using the nsP3 and PRDX1 antibodies. The fourth immunoblot represents the negative control, where rabbit IgG was used to immunoprecipitate the protein complex and was probed with anti-nsP3 antibody.

One of the crucial molecular functions associated with the CHIKV-nsP3-identified interacting partners in the current study is peroxidase activity, and the protein involved includes peroxiredoxin (PRDX1), with unique peptides equal to three and a peptide sequence represented in Figure 7a. As a first step, we checked for the co-localization pattern of the two proteins, i.e., CHIKV-nsP3 and PRDX1, using an immunofluorescence assay. The result showed that the two proteins were colocalized together in cytosol but the colocalizing signal was minimal, with a Pearson coefficient of 0.15 (Figure 7b). Further, we generated a STRING network for the proteins associated with peroxidase activity using high-confidence criteria of 0.7 in the STRING setting, represented in Figure 7c. As a final validating step, we performed Co-IP to investigate the direct interaction of the two proteins. However, our attempts to pull down endogenous oxidative stress scavenger protein peroxiredoxin (PRDX1) in CHIKV-infected cells using the anti-nsP3 antibody, or vice versa, were not successful (Figure 7d).

### 2.9. Validation of Interaction Between Host Partner eIF2α and CHIKV-nsP3

**Figure 8:**
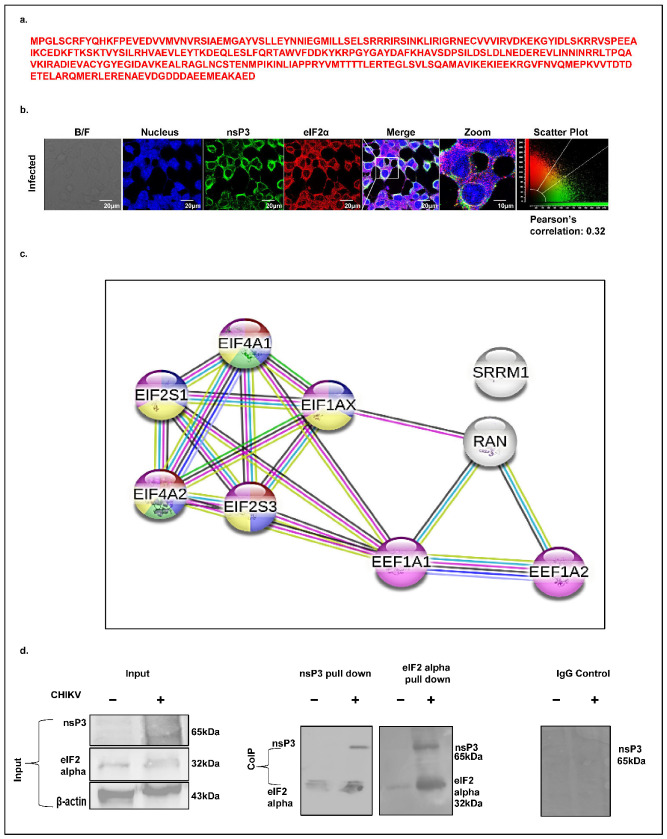
CHIKV-nsP3 protein interacts with eIF2α. (**a**) Peptide sequence of EIF2S3 identified through mass spectrometry. (**b**) Immunofluorescence showing the colocalization of eIF2α with CHIKV-nsP3 (at MoI of 1.0 in Huh7 cells). Immunostaining was carried out with antibodies against CHIKV-nsP3 (Green) and eIF2α (Red) and respective Alexa Fluor 488 (mice) and Alexa Fluor 594 (Rabbit) secondary antibodies. The nucleus was visualized with DAPI (blue). Images were taken using the confocal microscope. A scale bar of 20 μm was used. (**c**) STRING network represents the host proteins involved in host cell translation regulation. Nodes represent the host proteins involved during the translational process, and edges represent the association between the proteins based on similar functions. Filled nodes denote the prediction of the 3D structure of the specific protein. Light blue and purple edges represent the known interactions that have been experimentally validated, whereas green, red, and dark blue denote the predicted interactions. (**d**) Immunoblotting image showing the direct interaction between eIF2α and CHIKV-nsP3 during infection. The first blot represents the total cellular lysate (input) of the protein used for the pull-down assay and immunoblotting. The second and third immunoblot is the co-immunoprecipitation analysis showing the interaction of CHIKV-nsP3 protein with eIF2α using the nsP3 and PRDX1 antibodies. The fourth immunoblot represents the negative control, where rabbit IgG was used to immunoprecipitate the protein complex and was probed with anti-nsP3 antibody.

Eukaryotic translation initiation is tightly regulated by multiple eukaryotic initiation factors (eIFs), among which eIF2α (EIF2S1) plays a central role. A mass spectrometry analysis identified eIF2α as a CHIKV-nsP3-interacting host factor, supported by the presence of three unique peptides (Figure 8a). Given that eIF2α phosphorylation is a central event in stress response and translation inhibition, we examined its spatial association with CHIKV-nsP3. A confocal immunofluorescence analysis of CHIKV-infected Huh7 cells (12 hpi, MOI 1) revealed limited cytosolic co-localization between eIF2α and nsP3, with a Pearson correlation coefficient of 0.30 (Figure 8b). To contextualize eIF2α within the broader translation regulatory network, we generated a high-confidence STRING interaction map (confidence score ≥ 0.7). The resulting network, enriched for protein–protein interactions, comprised 9 nodes and 18 edges, including key translation factors such as EIF2S1, EIF2S3, EIF4A1, EIF4A2, and EIF1AX (Figure 8c). To validate this interaction biochemically, we performed co-immunoprecipitation of nsP3 and eIF2α from CHIKV-infected cell lysates at 12 hpi, followed by a immunoblot analysis. The results confirmed a association between nsP3 and eIF2α during infection (Figure 8d), reinforcing its potential role in modulating host translation pathways to favor viral replication.

## 3. Discussion

Our study provides a comprehensive analysis of the CHIKV-nsP3 interactome in human liver-derived Huh7 cells, identifying 52 statistically significant host interacting partners through high-throughput Co-IP coupled with LC-MS/MS. By employing a stringent selection criterion with three biological replicates, we minimized nonspecific binding and ensured the reliability of our findings. The overlap between the host proteins identified in this study and those reported in the previous literature, such as stress granules (e.g., G3BP1/2) and heat shock proteins (e.g., HSP40 and HSP90), confirms the authenticity of these interactions in the CHIKV life cycle [22,29]. As expected, the analysis confirmed the presence of a few viral proteins in the infected samples, including nsP2, E1, and the capsid protein. Previous studies have established the interaction between CHIKV-nsP3 and nsP2, especially during the early stages of infection [19]. The detection of additional viral proteins may be attributed to active viral replication and their potential indirect interactions with other host proteins [21,30]. However, it is worth noting that to understand its functional aspect with nsP3, a high number of peptides of the structural protein capsid warrants additional studies in the future.

Our CHIK-nsP3 host interactome and STRING network analyses identified a set of commonly targeted cellular host proteins involved in the metabolic, translational, and immune signaling pathways—processes that are critical for cellular response to infection. For instance, the metabolic enzyme TP1 has been shown to facilitate the replication of white spot syndrome virus [31], while OAT, another member of the interactome, that is crucial in ornithine metabolism, has been implicated in SARS-CoV-2 disease progression [32]. Additionally, the TCA cycle metabolite succinate is known to inhibit influenza virus replication by promoting succinylation of the viral protein [33]. Some of the important host translational factors, such as eEF1A, are known to facilitate ubiquitin-mediated proteasomal degradation of M protein, thereby suppressing rhabdovirus replication [34]. Similarly, translation initiation factor eIF4E is known to serve as an important host target for several viruses, regulating the translation of viral mRNAs [35]. We also identified the calcium-binding protein S100A, which has been reported to interact with the nsP3 protein of EEEV in NIH3T3 cells [28].

A key finding of this study is the enrichment of nsP3 interactors involved in cellular metabolism, particularly pyruvate metabolism and amino acid biosynthesis. The host glycolytic enzyme pyruvate kinase M (PKM), identified as a direct interactor of nsP3 (validated by IP and co-localization with a Pearson coefficient of 0.44), may contribute to ATP generation within the viral replication complex. This ATP could support the activity of other nsP3 interactors, such as DEAD-box helicases DDX5 and DDX17, which require energy for RNA unwinding to facilitate rapid viral RNA synthesis [36]. PKM’s role in viral replication is not unique to CHIKV; it interacts with the influenza virus RNA-dependent RNA polymerase [37] and modulates dengue virus (DENV) infection by facilitating exocytic virus release [38]. Additionally, PKM2, a PKM isoform, interacts with the classical swine fever virus (CSFV) NS4A protein, inducing mitophagy via the AMPK-mTOR pathway to enhance replication [39]. These findings suggest that PKM is a conserved viral target across diverse viral families, making it a potential candidate for host-directed therapies.

Another significant interactor, argininosuccinate synthase 1 (ASS1), a rate-limiting enzyme in arginine metabolism, represents a novel finding in the context of CHIKV–host interactions. Our IP and confocal microscopy data (Pearson coefficient: 0.76) confirm a stable cytosolic interaction between nsP3 and ASS1 at 12 hpi. While ASS1′s role in cancer is well-documented, its involvement in viral infections is emerging. Recent studies have shown ASS1 interactions with the capsid protein of Coxsackievirus B3 (CVB3) in macrophages [40] and its regulation of herpes simplex virus (HSV) and human cytomegalovirus (HCMV) infections [41]. We hypothesize that ASS1 may regulate arginase 1 (ARG1), another interactor identified in our study, to promote ornithine production, a precursor of polyamines that enhance viral replication. ARG1′s role in RNA viruses like CHIKV, coronaviruses, and hepatitis C virus (HCV) is well-established [42,43,44], supporting the plausibility of this mechanism in CHIKV infection. Interestingly, in our proteomics data, we have also identified a host factor, i.e., arginyl tRNA synthetase 1, which possesses an important regulatory role when the cellular arginine level is compromised due to inflammation [45]. This further strengthens our hypothesis, highlighting the importance of arginine during CHIKV infection in liver cells.

This study further highlights the manipulation of host translational machinery by CHIKV, with nsP3 interacting with eukaryotic initiation factors (eIFs) such as eIF2α (validated by IP and co-localization, Pearson coefficient: 0.30). eIF2α is a critical regulator of translation initiation, and its phosphorylation typically inhibits global translation to favor antiviral responses like stress granule formation. However, CHIKV’s ability to suppress stress granule assembly via nsP3-G3BP1/2 interactions [29] may be complemented by its interaction with eIF2α, potentially preventing phosphorylation and sustaining viral protein synthesis. The dynamic localization of eIF2α, shifting from cytosolic co-localization with nsP3 at 12 hpi to nuclear localization by 24 hpi, suggests the temporal regulation of this interaction, possibly linked to viral replication and assembly stages. This aligns with observations in other alphaviruses, such as Sindbis and Semliki Forest viruses, which inhibit host translation to suppress antiviral responses [46,47]. Viruses often exploit host translational machinery to prioritize their protein synthesis while suppressing innate immune defenses, and eIF2α serves as a regulatory hub in this ongoing battle [48].

Our analysis also identified peroxiredoxin 1 (PRDX1) as a secondary interactor of nsP3, though we were unable to validate its direct interaction or co-localization. PRDX1, an oxidative stress scavenger, plays diverse roles in viral infections, promoting antiviral innate immunity in pseudorabies virus (PRV) via the type I IFN pathway [49] and supporting influenza virus replication by interacting with ribonucleoproteins [50]. We hypothesize that PRDX1 may regulate innate immunity during CHIKV infection, a possibility that requires further exploration given its failure to be validated in our assays, potentially due to low-affinity interactions or context-specific associations.

**Figure 9:**
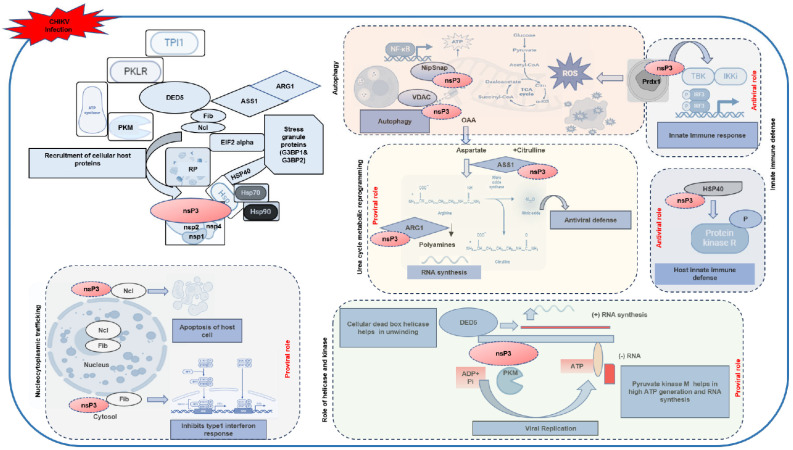
Schematic representation of CHIKV-nsP3 interactions with host proteins and their functional implications during infection. This hypothetical model summarizes the cellular pathways and processes modulated by CHIKV-nsP3 based on proteomic analysis and experimental validation. CHIKV-nsP3 (shown in red ovals) interacts with host proteins across multiple functional domains (host proteins are shown in blue color around the replication complex with no dashed rectangle boundary). In the cytoplasm, nsP3 recruits host translational machinery (EIF2α and DDX5), metabolic enzymes (PKM, PKLR, and TP1), stress granule proteins (G3BP1/2), and chaperones (HSP70 and HSP90) to facilitate viral replication. nsP3 modulates mitochondrial and redox homeostasis via interactions with VDAC, NipSnap, and Prdx1, influencing ROS production and autophagy. In metabolic reprogramming, nsP3 redirects the urea cycle through ARG1 and ASS1 to promote polyamine synthesis and viral RNA replication while dampening antiviral metabolic outputs. Interactions with innate immune modulators such as Prdx1, HSP40, and PKR suggest inhibition of the type I interferon response. Nuclear interactions with Nucleolin (Ncl) and Fibrillarin (Fib) may interfere with nucleocytoplasmic trafficking and promote apoptosis or inhibit the interferon response. In addition, nsP3 partners with PKM and DDX5 to ensure sufficient ATP generation and RNA unwinding necessary for efficient genome replication. The schematic presents these pathways as independent, context-specific processes, with nsP3 depicted at multiple sites to illustrate its distinct roles in each. Arrow thickness and color represent interaction directionality and functional outcomes (proviral or antiviral), and duplicated elements (e.g., nsP3 and HSP40) are used to reflect involvement across pathways.

Taking all the evidences obtained in the analyses performed in this study, we generate a hypothetical working model of the roles of the identified host interacting partners in the CHIKV life cycle (Figure 9). At the core of the model, CHIKV infection triggers the recruitment of host proteins by nsP3, a multifunctional viral protein known to orchestrate replication and host manipulation [30]. CHIKV-nsP3 is known to participate, along with other CHIKV nonstructural proteins (nsP1, nsP2, and nsP4), within the replication complex, in forming a hub for viral RNA synthesis. Host proteins such as nucleolin (Ncl) and fibrillarin (Fib) are recruited from the nucleus to the cytosol, a process indicative of nucleo-cytoplasmic shuttling. Nucleolin has been implicated in viral infections, such as SARS-CoV-2, where it interacts with viral proteins to induce apoptosis via p53 [51]. Similarly, fibrillarin aids viral entry in macrophages by suppressing type I interferon (IFN) responses [52]. In CHIKV infection, these nucleolar proteins may inhibit type I IFN responses, as shown in the model, thereby facilitating viral replication by dampening host antiviral defenses. The model further illustrates CHIKV’s impact on mitochondrial dynamics, with nsP3 interacting with proteins like VDAC, NIPSNAP, and those involved in the TCA cycle (e.g., succinate dehydrogenase and ATP citrate lyase). These interactions disrupt mitochondrial homeostasis, leading to increased ROS production and altered energy metabolism, which support viral replication while impairing antiviral signaling via MAVS [53]. VDAC’s role in viral infections, such as dengue and Japanese encephalitis virus (JEV), involves altering mitochondrial membrane potential [54,55]. The model also indicates that nsP3 induces autophagy, a process that may degrade host antiviral proteins, further facilitating viral persistence. Furthermore, our data suggest that CHIKV-nsP3 interacts with heat shock proteins (HSP40, HSP70, and HSP90) and protein kinase R (PKR), modulating cellular stress responses. HSP40, previously identified as a CHIKV interactor in macrophages [11], mediates PKR signaling, a critical regulator of host defense, as seen in influenza virus infections [56], suggesting that these interactions help CHIKV evade PKR-mediated antiviral responses, promoting viral replication.

This study addresses the knowledge gap in the role of host cellular mechanisms during CHIKV pathogenesis by employing a high-throughput interaction analysis to systematically identify novel interacting partners of CHIKV-nsP3 in human liver-derived cells, such as Huh7 hepatoma cells. Using techniques like LCMS, we aim to construct a comprehensive nsP3 interactome specific to hepatic cells, building on prior methodologies applied to other cell types [11]. The rationale is twofold: first, to elucidate the molecular mechanisms underlying CHIKV’s effects on the liver, an organ increasingly linked to clinical outcomes, and second, to identify potential therapeutic targets that could mitigate liver-specific damage or disrupt viral replication. Given the liver’s systemic importance and the paucity of data on CHIKV hepatic interactions, this research offers a novel perspective on viral pathogenesis.

The significance of this work lies in its potential to bridge clinical observations of liver dysfunction with molecular insights into CHIKV–host dynamics. By uncovering new nsP3 partners, we may identify pathways unique to liver cells, such as those tied to metabolism or immune signaling, that could be leveraged for host-directed therapies. This approach aligns with broader efforts to map host–virus interaction networks across tissues, enhancing our understanding of CHIKV’s systemic impact and informing strategies to combat both acute and chronic disease phases.

## 4. Materials and Methods

### 4.1. Cell Lines and Virus Infection

The hepatocellular carcinoma Huh7 cell line and African green monkey kidney-derived Vero cells (ATCC^®^ CCL-81™) were purchased from ATCC (Manassas, VA, USA). The human hepatoma-derived cells (Huh7) and African green monkey (Vero) kidney cells were maintained and propagated in Dulbecco’s minimal essential medium (DMEM, AL007A-500ML, purchased from Hichem life sciences, Thane, India) supplemented with 10% fetal bovine serum (FBS, RM10681-500ML, purchased from Hichem life sciences, Thane, India) and 100 U/mL penicillin and 100 µg/mL streptomycin antibiotics (A001A-100ML, purchased from Hichem life sciences, Thane, India) and in tissue culture flask with a 75 square centimeter growth surface. All the cells were incubated and propagated under standard conditions (37 °C, 5% CO_2_, and 95% humidity).

The CHIKV was isolated and purified from patient sera collected during an outbreak and propagated in both the C6/36 and Vero cell lines (Accession no JF950631.1) [57]. Huh7 cells were seeded and grown into 100 mm petri dishes in DMEM supplemented with 10% FBS. The cells were infected at a multiplicity of infection (MOI) of 1 with the CHIKV strain. The virus was diluted in sera and antibiotic-free DMEM and incubated for 2 h at 37 °C. The cells were washed with phosphate-buffered saline (PBS) 2 hpi and grown in complete medium. At different time points, the cells and media were collected. The cell pellets were washed with PBS and resuspended in a RIPA lysis buffer (freshly prepared in the lab) for immunoblot analysis.

### 4.2. Co-Immunoprecipitation Assay

The CHIKV-nsP3 protein was synthesized and purified according to protocols described in previous studies [11,58]. For antibody generation, the purified nsP3 protein was emulsified with Freund’s complete adjuvant (F5881, purchased from Sigma Aldrich Chemicals Private Limited, Bangalore, India) and used to immunize laboratory rabbits, following established procedures. The generated antibody was further purified using purified by protein-A and protein-G beads (GE17-0618-01, purchased from Sigma Aldrich Chemicals Private Limited, Bangalore, India) checked for its cross reactivity and specificity as previously reported. The raised anti-nsP3 antibody was further used for Co-IP experiments.

The in-house generated anti-nsP3 antibody was subsequently employed for co-immunoprecipitation (Co-IP) experiments to identify host cellular proteins interacting with CHIKV-nsP3. For this, lysates from CHIKV-infected Huh7 cells were incubated with the purified anti-nsP3 antibody using the Pierce co-immunoprecipitation kit (26149, purchased from Invitrogen Bioservices India Pvt.Ltd., Thermo Scientific, Haryana, India) as per the manufacturer’s protocol. Briefly, a CHIKV-nsP3-specific antibody was incubated with 100 µL of Protein A beads (provided with the kit) at 4 °C overnight. Next, an unbound antibody was washed off, and the antibody-labeled beads were further incubated with an infected Huh7 cell lysate (1 mg of total protein concentration) overnight at 4 °C. Subsequently, the beads were washed with an IP wash buffer (provided with the kit), followed by three sets of elutions using an elution buffer (provided with the kit). The Co-IP elutions were subjected to mass spectrometry analysis.

### 4.3. Sample Preparation for Mass Spectrometry

The Co-IP eluates were subjected to in-solution trypsin digestion. Firstly, elutes were adjusted with 50 mM ammonium bicarbonate, pH 8.0, followed by reduction with 10 mM Dithiothreitol (DTT) (D9779, purchased from Sigma Aldrich Chemicals Private Limited, Bangalore, India) for an hour at RT. Next, it was alkylated with 40 mM iodoacetamide (IAA) (SML4172, purchased from Sigma Aldrich Chemicals Private Limited, Bangalore, India) for an hour. Using trypsin-Gold (Promega, Madison, WI, USA) at a 1:50 ratio, the digestion of protein eluates was performed via incubation in a water bath at 37 °C for 16 h. The trypsin digestion reaction was stopped using formic acid (0.1%) (F0507, purchased from Sigma Aldrich Chemicals Private Limited, Bangalore, India). The digested peptides were concentrated in a speed-vac and analyzed by LC-MS/MS (Vproteomics Pvt Ltd., New Delhi, India).

### 4.4. Mass Spectrometry

The LC-MS/MS analysis was performed Orbitrap Velos Pro MS coupled to Easy n-LC 1000 (Thermo Fisher Scientific, Waltham, MA, USA). Tryptic digested peptide mixtures were first loaded onto a reverse-phase C18 pre-column (Acclaim PepMap, 75 mm × 2 cm, 3 mm, 100 A°, Thermo Fisher Scientific, Waltham, MA, USA) and then separated using an analytical column (Acclaim PepMap, 50 mm × 15 cm, 2 mm, 100 A°) using a gradient of 5% solvent B (0.1% formic acid in 95/5 acetonitrile/water) to 35% solvent B in 35 min. The eluted peptides were injected into the mass spectrometer, and MS1 data were acquired using a mass range from 350 to 2000 Da in full scan mode at 60,000 resolutions. The top 15 precursors were allowed to fragment using CID (collision-induced dissociation) in the ion trap, with a collision energy of 35. MS data was acquired using a data-dependent top 10 method, dynamically choosing the most abundant precursor ions from the survey scan. The raw data were analyzed using Proteome Discoverer (v2.2; Thermo Scientific, Waltham, MA, USA) (Proteome Discoverer Software|Thermo Fisher Scientific, Waltham, MA, USA) with the SEQUEST algorithm against the Uniprot database (UniProt; accessed on 2 April 2025). For Sequest search, the precursor and fragment mass tolerances were set at 10 ppm and 0.5 Da, respectively. The protease used to generate peptides, i.e., enzyme specificity was set for trypsin/P (cleavage at the C terminus of “K/R”: unless followed by “P”) along with a maximum missed cleavages value of two. Carbamidomethyl on cysteine as a fixed modification and oxidation of methionine and N-terminal acetylation were considered as variable modifications for database search. The identification of peptides was validated using both peptide spectrum match and a protein false discovery rate set to 0.01 FDR.

### 4.5. Data Analysis

LC-MS experiments were performed with three independent biological replicates to ensure the reproducibility and robustness of the results. The selection criteria of the host proteins were based on the protein abundance of both the viral (nsP3) and the host protein in all three infected biological replicates, and a false discovery rate (FDR) of 1% and a standard cutoff *p*-value of ≤0.05 were employed as the threshold for significance. Protein abundances were calculated as the average among three biological replicates of the summed intensities. Peptide groups were considered for quantification based on their uniqueness (unique peptides) and following the principle of parsimony.

### 4.6. Bioinformatics Analysis

The proteins/peptides satisfying high confidence were used in subsequent statistical analyses in XLSTAT (v2024.1; XLSTAT|Statistical Software for Excel; accessed on 5 April 2025). Common proteins between the CHIKV uninfected and infected libraries were excluded, and unique proteins with a FDR of 1% were further assessed for functional annotation, protein–protein interaction network construction, and subcellular localization. Multiple databases such as Metascape (v3.5.; Metascape, accessed on 28 March 2025), GProfiler (v0.2.2) (g:Profiler—a web server for functional enrichment analysis and conversions of gene lists), Shiny go (ShinyGO 0.82), Funrich (v3.1.3; FunRich :: Functional Enrichment Analysis Tool :: Home, accessed on 23 March 2025), and STRING 12 were used for a functional annotation analysis [59,60,61]. STRING functional annotation chart tools (v12.0; STRING: functional protein association networks; accessed on 8 April 2025) were used to perform the protein domain analysis. The proteins were classified into their respective pathways using different pathway analysis servers, i.e., KOBAS (v3.0; KOBAS; accessed on 3 March 2025) [62]. The multiple gene set testing and *p*-value adjustments were performed using the Benjamini–Hochberg test. Significant pathways were selected with corrected *p*-values < 0.05.

### 4.7. In Silico Subcellular Localization and Network Construction

The cellular component of the host proteins was analyzed using Funrich (v3.1.3) and STRING 12.0. Subcellular localization of the unique host proteins of nsP3-IP was identified using the web tool DeepLoc-1.0 (DeepLoc 1.0—DTU Health Tech—Bioinformatic Services; accessed on 3 April 2025) and by extracting the information from the Human Protein Atlas database (The Human Protein Atlas; accessed on 3 April 2025).

### 4.8. Protein–Protein Interaction Network of the CHIKV-nsP3 Interacting Partners

The STRING 12 database fetched the host–virus protein–protein interaction (PPI) network (STRING: functional protein association networks). To find the hub of proteins, the interactors identified through the Co-IP-based LCMS method were subjected to STRING database enrichment with an interaction score of 0.7 for each protein. The network was further visualized by Cytoscape (v3.10.3; Cytoscape: An Open Source Platform for Complex Network Analysis and Visualization; accessed on 11 April 2025). According to their organellar localization, the proteins in the network were grouped. Cytoscape (v3.10.3) was used to visualize the network.

### 4.9. Western Blot Analysis

The cells were harvested 12 h post-infection and lysed using a freshly prepared RIPA lysis buffer (freshly prepared) supplemented with protease inhibitor cocktails (PICs) (11697498001, complete(tm) protease inhibitor, purchased from Sigma Aldrich Chemicals Private Limited, Bangalore, India). The lysate was collected after centrifugation, and the protein concentration was estimated using the BCA reagent (23225, Invitrogen Bioservices India Pvt.Ltd, Thermo Scientific, Haryana, India). Protein samples in equal concentration were prepared in 6× SDS loading dye (prepared freshly in the lab) and denatured at 95 °C for 15 min. The proteins were resolved on 10% SDS-PAGE (prepared freshly), followed by Western transfer of the proteins onto a nitrocellulose membrane (1620112, Bio-Rad, Delhi, India). The membrane was then blocked with 5% BSA (10735086001, BSA fraction v, 100G purchased from Sigma Aldrich Chemicals Private Limited, Bangalore, India) prepared in 1× PBS for 1 h at room temperature. Subsequently, the membrane was incubated with primary antibodies (1:2000 dilution) prepared in 2.5% BSA and incubated at 4 °C overnight. The primary antibodies used in the study were lab-generated CHIKV anti-nsP3 (mice and rabbit), and CHIKV E1 (mice) [11,58,63]. The ASS1 monoclonal antibody used for the Western blotting experiments was purchased from Cell Signaling Technology (D404B), Imperial Life Sciences (P) Ltd., Gurugram, India. The β-actin antibody (sc-47778) was purchased from BI BIOTECH INDIA PVT. LTD. (New Delhi, India). The PKM (3186S), PRDX1 (D5G12), and eIF2α (D7D3) monoclonal antibodies were purchased from Cell Signaling Technology (Gurugram, India). The next day, the membrane was washed three times with 1× PBST for 10 min each and then incubated with HRP-conjugated secondary antibody (1:5000 dilution) in 2.5% BSA + PBST for 1 h at RT. The anti-mouse (NB120-6808) and anti-rabbit HRP-conjugated (A6154-1ML) secondary antibodies were purchased from Novus 567 and Sigma Aldrich Chemicals Private Limited (Bangalore, India), respectively. The membrane was then washed thrice with 1× PBST for 10 min each and visualized in Chemidoc MP (Bio-Rad, Delhi, India).

### 4.10. Confocal Immunofluorescence Microscopy

Huh7 cells were seeded and grown on a sterile glass coverslip in a 6-well culture plate. The next day, the cells were infected in serum-free DMEM (DMEM, AL007A-500ML, Hichem Life Sciences, Thane, India) with CHIKV for 2 h, and the serum-free medium was replaced with 2% DMEM and incubated for 12 hours. The cells were then washed with ice-cold 1× PBS (freshly prepared) and subsequently fixed and permeabilized with 4% paraformaldehyde (F8775-500ML, FORMALDEHYDE MOLECULAR BIOLOGY, Sigma Aldrich Chemicals Private Limited, Bangalore, India) for 30 min and chilled methanol (100%) (Sigma Aldrich Chemicals Private Limited, Bangalore, India) at 4 °C for 1 h, respectively. Next, the cells were washed with 1× PBS and incubated with 1% BSA and 0.3% Triton X-100 (T8787, Sigma Aldrich Chemicals Private Limited, Bangalore, India) prepared in 1× PBS for 45 min. After blocking, the cells were incubated with primary antibodies (in-house generated and commercial, 1:100 dilution) overnight at 4 °C, followed by incubation with Alexa Fluor 488 and 594 conjugated secondary antibodies (Abcam, New Delhi, India) after washing with 1× PBS for 1 h at room temperature. Alexa Fluor^®^ 488 Anti-mouse (ab150113) and Alexa Fluor^®^594 Anti-Rabbit (ab150080) were purchased from Abcam (New Delhi, India). The cells were then washed with 1× PBS thrice, and nuclear chromatin was stained with DAPI dye (Invitrogen Bioservices India Pvt.Ltd., Haryana, India). Lastly, the coverslips were mounted on clean glass slides and visualized under a Nikon A1R laser scanning confocal microscope (Nikon India Private Limited, Gurugram, India). For the acquisition of the image, the settings such as laser power, voltage gain, and offset were decided for unstained samples to avoid the autofluorescence signal. 

### 4.11. Statistical Analysis

Experiments were conducted in triplicate, and the data were expressed as the mean standard deviation. A statistical analysis of the experimental data was performed using GraphPad Prism 6 (v6.0). A two-way analysis of variance with Tukey’s multiple comparison test was used for the plaque assay data analysis. Significant differences were denoted as follows: ns, nonsignificant; * *p* < 0.05; ** *p* < 0.01; and *** *p* < 0.001. Significant values (*p*-value < 0.05) are represented with asterisks.

## 5. Limitations

Our study identifies several novel CHIKV-nsP3 interacting host proteins, including ASS1, PKM, and eIF2α, alongside other factors previously implicated in viral life cycles. However, some of the limitations should be acknowledged. First, the nsP3-specific antibody used in the co-immunoprecipitation experiments displayed partial degradation and nonspecific binding, which could introduce artifacts. To mitigate this concern, we performed reverse co-immunoprecipitation using antibodies specific to selected host proteins, thereby strengthening the validity of the interactions. Despite these efforts and preliminary findings, further validation is necessary with respect to the in vitro study under rigorously controlled conditions and transfection-based experiments. In particular, future studies should aim to delineate the specific domains within nsP3 responsible for binding to key host factors such as PKM, ASS1, and eIF2α. Mutational analyses of nsP3 could provide mechanistic insights into the functional significance of these interactions during CHIKV infection. Additionally, while our Co-IP-based proteomic approach identified stable protein–protein interactions, it may not fully capture transient or dynamic interactions, nor detect critical post-translational modifications. Such layers of regulation are likely to be important in the context of CHIKV pathogenesis and warrant further investigation.

## Figures and Tables

**Table 1:** A list of all viral and host-interacting proteins of CHIKV-nsP3 identified through mass spectrometry and relevant studies in the case of other alphaviruses.

	Gene Stable ID	Gene Name	Gene Description	References (Alphavirus)
Viral proteins	CHIKV nsP3	NSP3	Nonstructural protein 3	this study
	CHIKV nsP2	NSP2	Nonstructural protein 2	[19]
	CHIKV E1	E1	Envelope protein	this study
	CHIKV Capsid	Capsid	Capsid protein	this study
Host proteins	ENSG00000149257	SERPINH1	serpin peptidase inhibitor	this study
	ENSG00000067225	PKM	pyruvate kinase M	this study
	ENSG00000134333	LDHA	lactate dehydrogenase	this study
	ENSG00000152234	ATP5A1	ATP synthase, H+ transporting, mitochondrial F1 complex, alpha subunit 1	[20]
	ENSG00000145907	G3BP1	GTPase activating protein	[14]
	ENSG00000115484	CCT4	chaperonin containing TCP1, subunit 4	[21]
	ENSG00000145349	CAMK2D	calcium/calmodulin-dependent protein kinase II delta	this study
	ENSG00000080824	HSP90	heat shock protein 90kDa	[22]
	ENSG00000128050	PAICS	phosphoribosylaminoimidazole carboxylase, phosphoribosylaminoimidazole succinocarboxamide synthetase	this study
	ENSG00000237289	CKMT1B	creatine kinase	this study
	ENSG00000110700	RPS13	ribosomal protein S13	this study
	ENSG00000150768	DLAT	dihydrolipoamide S-acetyltransferase	this study
	ENSG00000120438	TCP1	t-complex 1	[21]
	ENSG00000142864	SERBP1	SERPINE1 mRNA binding protein 1	this study
	ENSG00000167005	NUDT21	nudix	this study
	ENSG00000161970	RPL26	ribosomal protein L26	this study
	ENSG00000171858	RPS21	ribosomal protein S21	this study
	ENSG00000101182	PSMA7	proteasome	this study
	ENSG00000169710	FASN	fatty acid synthase	[23]
	ENSG00000142676	RPL11	ribosomal protein L11	this study
	ENSG00000116288	PARK7	Parkinson protein 7	this study
	ENSG00000087365	SF3B2	splicing factor 3b, subunit 2, 145kDa	this study
	ENSG00000101210	EEF1A2	eukaryotic translation elongation factor 1 alpha 2	[24]
	ENSG00000165704	HPRT1	hypoxanthine phosphoribosyltransferase 1	this study
	ENSG00000254772	EEF1G	eukaryotic translation elongation factor 1 gamma	this study
	ENSG00000092199	HNRNPC	heterogeneous nuclear ribonucleoprotein C	[25]
	ENSG00000108654	DDX5	DEAD Asp-Glu-Ala-Asp) box helicase 5	[26]
	ENSG00000143870	PDIA6	protein disulfide isomerase family A, member 6	[27]
	ENSG00000132507	EIF5A	eukaryotic translation initiation factor 5A	this study
	ENSG00000131473	ACLY	ATP citrate lyase	this study
	ENSG00000100028	SNRPD3	small nuclear ribonucleoprotein D3 polypeptide 18 kDa	this study
	ENSG00000099622	CIRBP	cold inducible RNA binding protein	this study
	ENSG00000113643	RARS	arginyl-tRNA synthetase	this study
	ENSG00000114942	EEF1B2	eukaryotic translation elongation factor 1 beta 2	this study
	ENSG00000130741	EIF2S3	eukaryotic translation initiation factor 2, subunit 3 gamma, 52 kDa	this study
	ENSG00000168291	PDHB	pyruvate dehydrogenase	this study
	ENSG00000176974	SHMT1	serine hydroxymethyltransferase 1	this study
	ENSG00000171314	PGAM1	phosphoglycerate mutase 1	this study
	ENSG00000115053	NCL	nucleolin	this study
	ENSG00000118520	ARG1	arginase 1	this study
	ENSG00000092621	PHGDH	phosphoglycerate dehydrogenase	this study
	ENSG00000130707	ASS1	argininosuccinate synthase 1	this study
	ENSG00000187109	NAP1L1	nucleosome assembly protein 1-like 1	[16]
	ENSG00000103423	DNAJA3	DnaJ	[11]
	ENSG00000143106	PSMA5	proteasome	this study
	ENSG00000143556	S100A7	S100 calcium binding protein A7	[28]
	ENSG00000065154	OAT	ornithine aminotransferase	this study
	ENSG00000117450	PRDX1	peroxiredoxin 1	this study
	ENSG00000147140	NONO	non-POU domain containing, octamer-binding	this study
	ENSG00000105202	FBL	fibrillarin	this study
	ENSG00000134001	EIF2S1	eukaryotic translation initiation factor 2, subunit 1 alpha, 35 kDa	this study
	ENSG00000111669	TPI1	triosephosphate isomerase 1	this study

**Table 2:** A list of all the host-interacting proteins of CHIKV-nsP3 and the significant pathways analyzed using KOBAS v3.0 (pathway databases used were KEGG and Reactome) with *p* value < 0.05 (Benjamini–Hochberg test).

Pathways	Input	*p*-Value	Proteins
Ribosome	22	5.59 × 10^−23^	RPSA, RPL10A, RPL5, RPL9, RPL11, RPL19, RPL24, RPL26, RPL30, RPL27A, RPLP0, RPS2, RPS3, RPA3, RPS3A, RPS5, RPS13, RPS15A, RPS16, RPS20, RPS21, RPL36
Carbon metabolism	12	2.51 × 10^−11^	ALDOC, DLAT, OGDH, PDHB, PGAM1, PKLR, DLST, SDHA, PKM, TPI1, SHMT1, PHGDH
Glycolysis	9	1.12 × 10^−9^	PKLR, PDHB, TP1, DLAT, PGAM1, ALDOC, LDHA, LDHB, PKM
Biosynthesis of amino acids	9	2.44 × 10^−9^	PKLR, TP1, PHGDH, PGAM1, ALDOC, ASS1, SHMT1, PKM, ARG1
Spliceosome	10	2.05 × 10^−8^	CIRBP, DDX5, SNRPA1, SNRPB, SNRPE, SF3A2, SRRM1, DDX17, SF3B2, NUDT21
Citrate cycle (TCA cycle)	6	7.55 × 10^−8^	OGDH, DLST, DLAT, SDHA, ACLY, PDHB
Metabolic pathways	27	1.82 × 10^−7^	CKMT1A, CKMT1B, ATP5B, ASS1, OAT, PKM, PKLR, OGDH, HPRT1, PAICS, FASN, EPRS, PHGDH, PDHB, TP1, DLAT, PGAM1, ALDOC, LDHA, LDHB, SHMT1, DLST, GPX5, SDHA, ACLY, ATP5A1, CKB
RNA transport	9	1.22 × 10^−6^	RAN, EEF1A1, EEF1A2, EIF1AX, SRRM1, EIF4A1, EIF2S1, EIF2S3, EIF4A2
Pyruvate metabolism	5	6.88 × 10^−6^	DLAT, PDHB, LDHA, LDHB, PKM
Arginine and proline metabolism	4	2.09 × 10^−5^	ARG1, ASS1, OAT, PKM
Huntington disease	8	2.72 × 10^−5^	TGM2, ATP5A1, CLTA, CLTC, ATP5B, SDHA, VDAC2, VDAC1
Glucagon signaling pathway	6	6.35 × 10^−5^	CAMK2D, PGAM1, PDHB, LDHA, LDHB, PKM
HIF-1 signaling pathway	5	7.72 × 10^−5^	CAMK2D, ALDOC, LDHA, LDHB, PDHB
Parkinson disease	6	3.46 × 10^−5^	ATP5A1, ATP5B, SHDA, VDAC2, VDAC1, PARK7
Protein processing in endoplasm	5	6.94 × 10^−4^	PD1A6, HSP90AA1, EIF2S1, CAPN1, SKP1
IL-17 signaling pathway	3	7.25 × 10^−3^	HSP90A1, S100A7, S100A8
Ferroptosis	2	1.28 × 10^−2^	VDAC2, PCBP2

## Data Availability

All relevant data are reported within this paper and its Supporting Materials.

## Supplementary Materials

The supporting information can be downloaded at https://www.mdpi.com/article/10.3390/ijms26146832/s1. References [11,58] are cited in the Supplementary Materials.

## Abbreviations

Co-IP—co-immunoprecipitation; CHIKV—chikungunya virus; CHIKD—chikungunya disease; nsP3—nonstructural protein 3; ASS1—argininosuccinate synthase 1; PKM—pyruvate kinase M; Prdx1—peroxiredoxin; eIF2α—elongation initiation factor 2 alpha subunit.
