# Supplementary material for: Proteomic Analysis of CHIKV-nsP3 Host Interactions in Liver Cells Identifies Novel Interacting Partners
Source: Int J Mol Sci. 2025 Jul 16;26(14):6832. doi: 10.3390/ijms26146832 (PMC12294820; doi:10.3390/ijms26146832)
Supplement: Supplementary file 1 [file ijms-26-06832-s001.zip › ijms-3661642-supplementary.pdf]

Proteomic Analysis of CHIKV-nsP3 Host Interactions in Liver Cells Identifies Novel Interacting Partners

Nimisha Mishra[1,2], Yash Chaudhary[1#], Sakshi Chaudhary[1], Anjali Singh[1], Priyanshu Shrivastava [1,@], Sujatha Sunil[*1]

[1] International Centre for Genetic Engineering and Biotechnology, New Delhi

[2]TERI School of Advanced Studies (TERI-SAS), New Delhi

[#]Current Address: Department of Microbiology and Plant Pathology, University of California, Riverside, California, USA

[@] Currently working in the Department of Hematopoietic Biology and Malignancy, MD Anderson Cancer Center, Houston, TX, USA

Corresponding author

Sujatha Sunil: sujatha@icgeb.res.in

Orcid Identifier: 0000-0002-6531-7768

S1. Characterization of CHIKV nsP3 antibody in Huh7 cells

CHIKV nsP3 protein was purified based on methods outlined in previous publications, and antibody was raised in a rabbit. (Kumar et al., 2021; Srivastava et al., 2024).

The raised antibody was purified with Protein A and Protein G beads. Further, we performed western blotting in infected Huh7 lysate to check the cross-reactivity and efficacy of the antibody. The result showed a single band of 65 kDa nsP3 protein size during infection, and uninfected lysate served as a control (shown in Fig. S1a). We also optimized the specificity of the raised antibody before performing CoIP experiments (shown in Fig. S1 b). Before carrying out the CoIP experiment, we optimized the specificity of the antibody in infected lysate and used IgG agarose beads as a negative control to filter the binding of non-specific proteins both in uninfected and infected samples. CoIP experiments were performed using a purified antibody.

Apart from this, we also checked for the localization pattern of CHIKV nsP3 in infected Huh7 cells using an immunofluorescence assay. The result showed the expression of CHIKV nsP3 in the cytosol (Fig.S1 c).

Taken altogether, the CHIKV nsP3 antibody shows a band size of 65kDa in Huh7 cells, and its localisation is cytosolic during infection.

Supplementary Information file

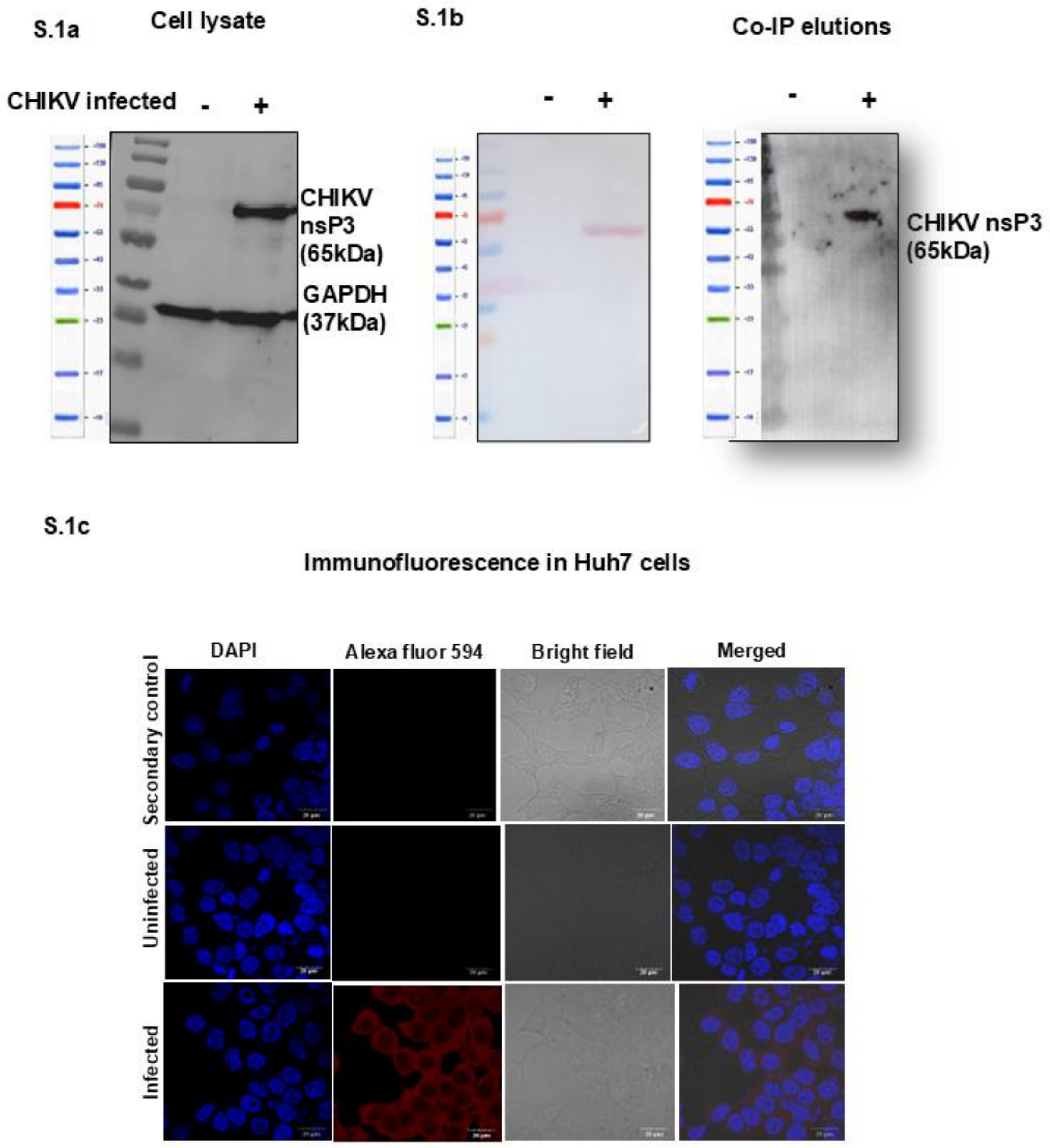

Figure S1: Validation of CHIKV nsP3 antibody a.) Validation of CHIKV nsP3 antibody in Huh7 lysate through western blotting b.) Validation of the nsP3 antibody in CoIp elutions (CHIKV infected lysate) c.) Immunofluorescence assay of CHIKV infected Huh7 cells using CHIKV nsP3 in house generated antibody. Huh7 cells were infected with CHIKV at MOI of 1 and fixed at 12 hpi with uninfected and secondary control. The cells were then incubated with anti-CHIKV nsP3 rabbit sera, followed by Alexa 594 anti-rabbit antibody. The nucleus was stained with DAPI. Bar length=20 µm.
